# Applying Essential Public Health Functions in building health systems’ resilience globally: Yu Zhang

**DOI:** 10.1093/eurpub/ckac129.205

**Published:** 2022-10-25

**Authors:** Y Zhang, S Mustafa, G McDarby, R Seifieldin, S Saikat, G Schmets

**Affiliations:** Health Services Resilience Team, WHO, Geneva, Switzerland

## Abstract

**Background:**

The COVID-19 pandemic has exposed Public Health system weaknesses due to chronic underinvestment in Public Health. In this context, Essential Public Health Functions (EPHFs) have been revitalized as an integrated, cost effective and sustainable approach to operationalising Public Health. The World Health Organization's recent position paper on building health systems resilience towards universal health coverage and health security recommended investing in EPHFs as a key mean for countries’ health systems recovery and transformation during COVID-19 and beyond. There is a need for conceptual and operational clarity of EPHFs to support countries to build back better, fairer and more resilient health systems.

**Methods:**

A rapid review of peer-reviewed and grey literature regarding the EPHFs was conducted to identify different actors’ understandings of EPHFs and key lessons of applying the EPHFs at the global, regional and national levels, in order to identify the added value and key enablers to operationalising EPHFs. A crosswalk analysis of different authoritative lists of EPHFs was conducted to develop a common list of EPHFs as a reference for countries in response to Public Health challenges.

**Results:**

A consolidated list of 12 EPHFs derived from the crosswalk analysis of different authoritative lists is presented, underpinning the consideration of health systems components and pressing health challenges. Six key enablers are identified from evidence and experience. These enablers are fundamental for countries to build holistic and strong Public Health capacities.

**Conclusions:**

The EPHFs provide a clear and integrated framing to operationalise Public Health in countries that can be adapted to country contexts to build resilience. Health authorities and other Public Health stakeholders must seize the opportunity brought by COVID-19 recovery to continue advocating for and strengthening Public Health as a priority in health systems’ reconstruction and reform.

